# Oxidative stress-induced 1, *N^6^*-ethenodeoxyadenosine adduct formation contributes to hepatocarcinogenesis

**DOI:** 10.3892/or.2013.2227

**Published:** 2013-01-04

**Authors:** LEI ZHOU, YUZHEN YANG, DEAN TIAN, YING WANG

**Affiliations:** 1 Department of Gastroenterology, Huazhong University of Science and Technology, Wuhan, Hubei 430030; 2 Institute of Liver Diseases, Tongji Hospital, Tongji Medical College, Huazhong University of Science and Technology, Wuhan, Hubei 430030; 3 Department of Gastroenterology, The Central Hospital of Wuhan, Tongji Medical College, Huazhong University of Science and Technology, Wuhan, Hubei 430014, P.R. China

**Keywords:** oxidative stress, 1, *N^6^*-ethenodeoxyadenosine, p53, hepatocarcinogenesis

## Abstract

Numerous studies have found that oxidative stress-derived 1, *N*^6^-ethenodeoxyadenosine (ɛ-dA) can act as a driving force towards hepatocellular carcinoma (HCC) in cancer-prone liver diseases. The aim of the present study was to determine the oxidative stress status and the occurrence of ɛ-dA in HCC and adjacent non-tumor liver tissue, and to clarify whether the occurrence of ɛ-dA is related to liver inflammatory activity, fibrosis and mutant p53 expression. Oxidative stress-related parameters were examined in tumor and (or) non-tumor liver tissues of 32 patients with HCC. ɛ-dA, mutant p53 and proliferating cell nuclear antigen (PCNA) were immunohistochemically investigated in control, HCC and non-tumor liver tissues. The total antioxidant capacity and total superoxide dismutase activity of HCC tissues were lower compared to those of non-tumor tissues (P<0.05 vs. P<0.001). The prevalence of ɛ-dA in HCC was significantly higher compared to control (P<0.0001) and non-tumor liver tissues (P<0.001). A significant correlation between the positive rate of ɛ-dA and mutant p53 was observed (r=0.5162, P<0.01). The positive rate of PCNA in HCC was significantly higher compared to control (P<0.0001) and non-tumor liver tissues (P<0.0001). There was a possible link between the formation of ɛ-dA and chronic inflammation and fibrosis. Therefore, ɛ-dA lesions may gradually accumulate in chronic liver diseases, and partially contribute to mutant p53 overexpression and excessive cell proliferation, making it a potential mechanism in oxidative stress-mediated hepatocarcinogenesis.

## Introduction

Hepatocellular carcinoma (HCC) is one of the most common types cancer in the world, accounting for 85–90% of the total primary liver cancer burden worldwide (1); it is also the third most frequent cause of cancer-related mortality (2). In China, HCC was projected to contribute more than 400,000 new cases and caused 370,000 deaths during 2008; in the same year, the incidence of HCC in American men and women was approximately 15,000 and 6,000 respectively (International Agency for Research on Cancer: http://www-dep.iarc.fr/). Therefore, HCC represents an international public health concern as one of the most common and aggressive types of cancer worldwide.

There are multiple etiological factors that are associated with the development of HCC, the most prevalent being chronic hepatitis B (HBV) and hepatitis C virus (HCV) infections (3); other non-viral causes of HCC include alcohol abuse, long-term exposure to aflatoxin B1 (AFB), iron overload syndromes, and non-alcoholic steatohepatitis (NASH).

The majority of HCC cases occur in fibrotic/cirrhotic livers, and the common mechanism for hepatocarcinogenesis is chronic inflammation associated with severe oxidative stress (4). There is a large body of evidence indicating that oxidative stress plays a common pathogenetic role in different agent-mediated hepatocarcinogeneses, including HBV (5,6), HCV (7,8), alcohol (9), AFB (10), NASH (11).

Oxidative stress results from a disruption of redox homeostasis due either to an elevation of reactive oxygen species (ROS) production or a decline of ROS-scavenging capacity (12). ROS are defined as molecules derived from oxygen with characteristic instability and chemical reactivity and include both free radicals, such as superoxide (O_2_^−^) and hydroxyl radical (OH^-^), and non-radicals, such as hydrogen peroxide (H_2_O_2_) and singlet oxygen (^1^O_2_). Cells are protected against ROS by scavenger enzymes such as superoxide dismutases (Mn-SOD and CuZn-SOD), and non-enzymatic antioxidants, predominantly glutathione (GSH). ROS can react with polyunsaturated fatty acids derived from membrane phospholipids or from dietary intake, resulting in the generation of lipid peroxidation (LPO) products such as *trans*-4-hydroxy-2-nonenal (4-HNE) and malondialdehyde (MDA) (13). 4-HNE can react with DNA bases such as deoxyadenosine and deoxycytidine to form the exocyclic etheno-DNA adducts including 1, *N*^6^-ethenodeoxyadenosine (ɛ-dA) and 3, *N^4^*-ethenodeoxycytidine (ɛ-dC) (14). These adducts are highly mutagenic; for example one leads to the missense mutations in the TP53 gene (which encodes p53) (15,16). Previous studies provided evidence that ɛ-DNA adducts may act as a driving force towards malignancy in cancer-prone liver diseases (9,17–19). However, detection of ɛ-DNA adducts in HCC and adjacent non-tumor liver tissues has not been reported.

Therefore, the aim of the present study was to: i) evaluate the status of oxidative stress in HCC and the surrounding non-tumor liver tissues; ii) determine the occurrence of ɛ-dA in these tissues; iii) determine if the formation of ɛ-dA was associated with liver inflammatory activity and fibrosis development; iv) assess if a correlation exists between the formation of ɛ-dA and mutant p53 expression in HCC tissue to elucidate its potential as a contributor to human hepatocarcinogenesis.

## Materials and methods

### Patients and liver specimens

We obtained tumor and (or) adjacent non-malignant liver tissues from 32 HCC patients who underwent hepatectomies at our hospital between 2010 and 2011. None of the patients had received chemotherapy or radiotherapy prior to surgery. Informed consent was obtained from all patients for subsequent use of their resected liver tissues. Tissue samples were collected immediately after liver resection. The non-malignant liver tissues were ≥2 cm in distance from the tumor margin. The paired tumors and adjacent non-malignant specimens were not always available for HCC. Half of the tissue was fixed in 4% paraformaldehyde and embedded in paraffin (30 patients had paired tumors and adjacent non-malignant tissues, from 2 patients only tumors were obtained). Serial sections (5 μm) were prepared for histopathological diagnosis and immunohistochemical staining. The other half of the tissue was washed extensively with 0.01 M phosphate-buffered saline (PBS; pH 7.4) solution to remove erythrocytes, and was then blotted on filter paper and stored at −80°C for further testing (28 patients had paired tumors and adjacent non-malignant tissues, from 2 patients only tumors were obtained and from 2 patients only non-malignant tissues). Additionally, control liver sections were obtained from 8 patients with benign space-occupying diseases of the liver who underwent surgery (5 liver hemangiomas, 1 hepatolithiasis, 1 focal nodular hyperplasia, 1 chronic granulomatous inflammation of the liver). The diagnoses were confirmed by histopathological study. Tumor staging was determined by the Tumor-Node-Metastasis (TNM) Classification (6th edit)of the International Union Against Cancer. Table I shows the general clinicopathological features of these 32 patients with HCC. The evidence of metastasis included vascular invasion, particularly to portal vein invasion and/or intrahepatic dissemination. Thirty HCC patients showed markers of hepatitis B virus infection, and 11 HCC patients had a history of alcohol abuse (mean alcohol consumption of 204.5±163.5 g/d; range 50–500 g/d). In 2 HCC cases, the underlying cause of the liver disease remained unknown. No HCV-related HCC was found in this study. The present study was performed according to the guidelines of the Ethics Committee of Tongji Hospital and was in accordance with the ethical standards of the World Medical Association Declaration of Helsinki.

### Histopathological examinations

Histopathological analyses were performed by H&E and Masson staining. Based on histological grade, tumors were subdivided into group I (well, well-moderately and moderately differentiated) and group II (moderately-poorly and poorly differentiated). The non-cancerous liver tissues were scored for the grade of necroinflammatory activity and stage of fibrosis according to the criteria of Desmet *et al*(20). Necroinflammatory activity (A) was graded according to the intensity of necroinflammatory lesions: 0, no histological activity; 1, mild activity; 2, moderate activity; 3, severe activity. The stage of fibrosis (F) was scored: 0, no fibrosis; 1, portal fibrosis without septa; 2, portal fibrosis with few septa; 3, numerous septa without cirrhosis; 4, cirrhosis. All the sections were analyzed without knowledge of clinical data.

### Analysis of oxidative stress-related parameters

Frozen tissue samples (100 mg) were mixed with 1.0 ml 0.01 M PBS (pH 7.4), incubated on ice and then homogenized. After centrifugation for 20 min at 12,000 rpm at 4°C, the supernatants were transferred to fresh tubes. After the protein concentrations were determined, GSH, total superoxide dismutase (SOD) activity, total antioxidant capacity (T-AOC) and MDA were assayed. GSH, total SOD activity and T-AOC of the liver tissue were detected with dithiobis-2-nitrobenzoic acid reactivity assay, xanthine oxidase-hydroxylamine method and ferric reducing ability of plasma method separately by using commercially available kits according to the manufacturer’s instructions (Jiancheng Biological Technical Institute, Nanjing, China). MDA levels were estimated with the thiobarbituric acid reactivity assay by using a commercially available kit according to the manufacturer’s instructions (Beyotime Biotech Inc., Haimen, China).

### Immunohistochemical detection of ɛ-dA

This was performed as previously described (18) with some modifications in our laboratory. The paraffin-embedded sections were dewaxed using xylene and hydrated in descending gradations of alcohol solutions, then washed in 0.01 M PBS (pH 7.4). The sections were treated in 3% H_2_O_2_ in absolute methanol for 10 min to block endogenous peroxidase activity. To enhance the immunostaining, sections were placed in citrate buffer (0.01 M, pH 6.0) and microwaved intermittently for up to 3 min at 800 W, 7 min at 640 W and 3 min at 480 W for antigen unmasking. Slides were slowly cooled down to room temperature. Slides were incubated with proteinase K (10 μg/ml) (Roche, Mannheim, Germany) to remove histone and non-histone proteins from DNA, increasing antibody accessibility. After washing with PBS, slides were treated with 20 μg/ml ribonuclease (RNAse; Solarbio Science & Technology Co., Ltd., Beijing, China) (heated for 10 min at 80°C to inactivate DNAse) at 37°C for 1 h to prevent antibody binding on RNA adducts, and then washed in PBS for 10 min. DNA was denatured by treatment with 4 N HCl for 5 min at room temperature and subsequently rinsed in PBS and double distilled water. The pH was neutralized with 50 mM Tris-base buffer, pH 7.4 for 5 min at room temperature. After washing with double distilled water and PBS, these sections were treated with 8% bovine serum albumin (BSA), 2% horse serum, 0.05% Tween-20 and 0.05% Triton X for 20 min at room temperature to block nonspecific binding sites. Slides were incubated with the primary monoclonal antibody EM-A-1 against ɛ-dA (diluted to 1:20, provided by Dr P. Lorenz and Dr M. Rajewsky, University of Essen, Germany) at 4°C overnight. After washing the slides with PBS for 10 min, antibody detection was performed using the Vectastain Elite ABC kit (Vector Laboratories, Burlingame, CA, USA), according to the manufacturer’s instructions. The slides were washed again and then the reaction products were visualized with diaminobenzidine (DAB; Dako, Glostrup, Denmark) as the chromogen. After stopping the reaction in H_2_O for 5 min, sections were counterstained with haematoxylin (Dingguo Changsheng Biotechnology Co., Ltd, Beijing, China) and covered with neutral balsam (Shanghai specimen and model factory, Shanghai, China). All sections were subjected to the same procedure under standardized conditions. Negative controls were performed by omitting the primary antibody.

### Immunohistochemical staining of p53, PCNA and α-SMA

Serial sections (5 μm) were prepared from paraffin blocks. Sections were deparaffinized and hydrated by sequential immersion in xylene and graded alcohol solutions, and finally washed in PBS (0.01 M, pH 7.4). The sections were then incubated in 3% H_2_O_2_ in absolute methanol for 10 min to quench endogenous peroxidase. Antigen retrieval was performed by microwaving the slides in citrate buffer (0.01 M, pH 6.0) for 3 min at 800 W, for 7 min at 640 W and for 3 min at 480 W. Slides were slowly cooled down to room temperature. After washing with PBS, these sections were treated with 8% BSA and 2% horse serum for 20 min at room temperature to block nonspecific binding sites. Slides were incubated with the primary antibodies overnight at 4°C. A mouse anti-p53 monoclonal antibody (diluted to 1:100; Boster Bioengineering Co., Ltd., Wuhan, China) was used to detect mutant p53 protein-positive cells. We used a mouse anti-PCNA monoclonal antibody (diluted to 1:400; Boster Bioengineering Co.) as the marker of cell proliferation. Immunohistochemical staining of α-smooth muscle actin (α-SMA) with a mouse monoclonal antibody (diluted to 1:150; Boster Bioengineering Co.) was performed to detect activated hepatic stellate cells (HSCs). After washing the slides with PBS for 10 min, antibody detection was performed using the Vectastain Elite ABC kit (Vector Laboratories), according to the manufacturer’s instructions. The slides were washed again and the reaction products were then visualized with DAB (Dako) as the chromogen. After stopping the reaction in H_2_O for 5 min, sections were counterstained with haematoxylin (Dingguo Changsheng Biotechnology Co.) and covered with neutral balsam. All sections were subjected to the same procedure under standardized conditions. Negative controls were performed by omitting the primary antibody.

### Imaging and semi-quantitative analysis of immunohistochemical staining

The frequency of ɛ-dA, mutant p53 and PCNA positively stained nuclei was expressed as % of stained cell nuclei over total number of cells counted, and 1,000 cells were observed in five or more random fields at a magnification of ×200 to calculate the percentage of positive cells.

### Statistical analysis

Data are expressed as the means ± standard deviation. Significant differences were calculated using the Student’s t-test or Mann-Whitney U test. Correlations were calculated by Spearman’s rank analysis. P<0.05 was considered to indicate statistically significant differences. All statistical analyses were performed using SPSS version 13.0 and GraphPad Prism version 5.0.

## Results

### Comparison of oxidative stress parameters in HCC and non-tumor liver tissues

**Figure 1:**
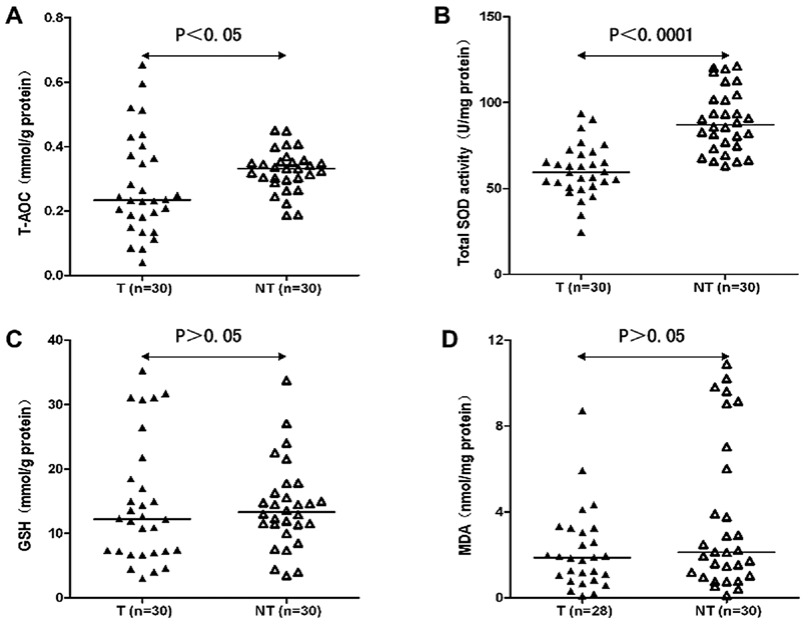
Comparison of oxidative stress parameters in HCC tissues (T) and non-tumor tissues (NT). (A) The T-AOC in T was lower than in NT. (B) The activity of total SOD in T was significantly lower than in NT. (C) The GSH content was not different between T and NT. (D) The content of MDA was not significantly different between T and NT. Black line represents median.

The T-AOC of HCC tissues (T) was lower compared to that of non-tumor liver tissues (NT) (0.277±0.156 mmol/g protein in T vs. 0.325±0.065 mmol/g protein in NT; P<0.05) (Fig. 1A), and the activity of total SOD also tended to be significantly lower in T when compared with the NT (60.420±15.222 U/mg protein in T vs. 89.381±18.064 U/mg protein in NT; P<0.0001) (Fig. 1B). The GSH content was not different between T and NT (14.595±9.531 vs. 14.140±6.699 mg/g protein; P>0.05) (Fig. 1C). The level of MDA was 2.200±1.884 nmol/mg protein in T, while it was 3.882±3.641 nmol/mg protein in NT, and the difference was not significant between them (P>0.05) (Fig. 1D).

### Relationship between T-AOC and total SOD activity in HCC tissues with clinicopathological features

As shown in Table II, to elucidate the biological significance of T-AOC and total SOD activity in HCC, we compared the levels of T-AOC and total SOD activity with the clinicopathological features of HCC patients. With respect to differentiation, we found that T-AOC and total SOD activity were significantly lower in group I than in group II (P<0.01 and P<0.05, respectively). On the other hand, it should be noted that the level of T-AOC was significantly higher in HCC cases with metastasis than without (P<0.05). Total SOD activity was not different between them (P<0.05). There were no significant differences regarding age, etiology, tumor number, TNM stage.

### Comparison of ɛ-dA prevalence in control liver, non-tumor liver and HCC tissues

**Figure 2:**
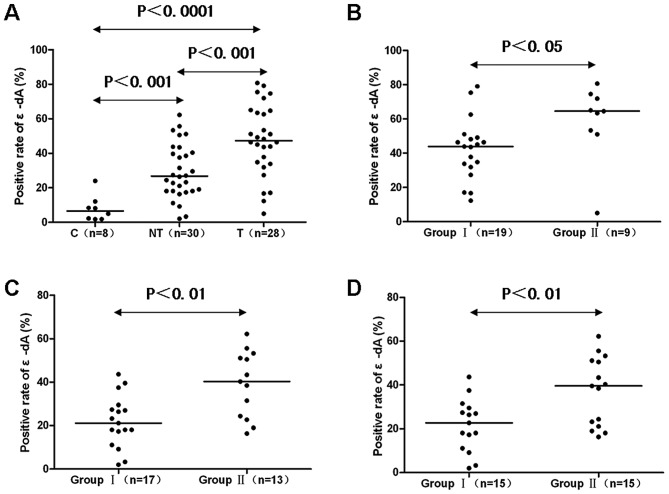
Comparison of ɛ-dA prevalence in liver tissues. (A) ɛ-dA prevalence in T was greater than in NT, and it was greater in NT compared to C. (B) The ɛ-dA prevalence in group II (moderately-poorly and poorly differentiated) HCC was greater than in group I (well, well-moderately and moderately differentiated) HCC. (C) Based on necroinflammatory activity, the positive rate of ɛ-dA in group II (moderate and severe chronic hepatitis) was higher than in group I (minimal and mild chronic hepatitis) NT. (D) Based on fibrosis degree, the positive rate of ɛ-dA in group II (severe fibrosis and cirrhosis) was higher than in group I (none and mild and moderate fibrosis) NT. C, control liver tissue; NT, non-tumor liver tissue; T, HCC tissue. Black line represents median.

**Figure 3:**
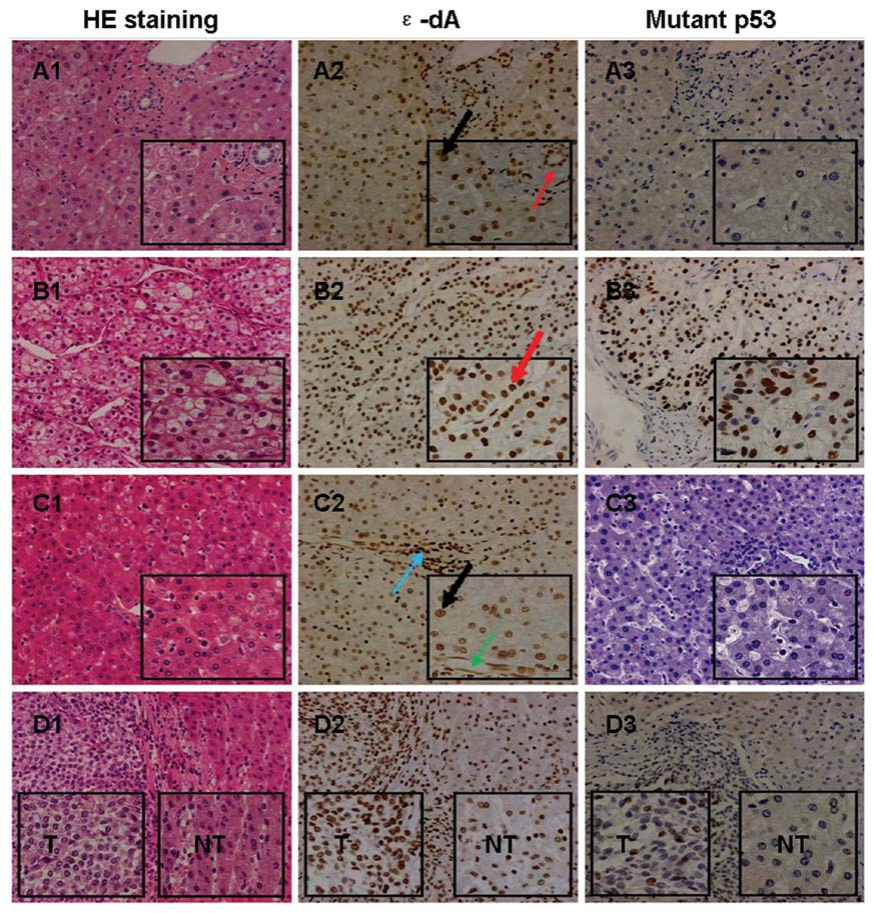
ɛ-dA and mutant p53 protein expression in liver tissues. (A) Patient no. 36: many ɛ-dA-positive cells (A2) were present in the adjacent liver tissue of chronic granulomatous inflammation (A1), but mutant p53 had a negative expression (A3). The thick black arrow shows the nuclear expression of ɛ-dA in hepatocytes; the thin red arrow shows the nuclear expression of ɛ-dA in bile duct epithelial cells. (B and C) Patient no. 39: many ɛ-dA-positive cells (B2 and C2) were present in HCC (B1) and non-tumor liver tissue (C1, >2 cm in distance from the tumor margin), and mutant p53 had a positive expression in HCC (B3), but a negative expression in non-tumor liver tissue (C3). The thick red arrow shows the nuclear expression of ɛ-dA in cancer cells; the thick black arrow shows the nuclear expression of ɛ-dA in hepatocytes; the thin blue arrow shows the nuclear expression of ɛ-dA in infiltrating lymphocytes; the thin green arrow shows the nuclear expression of ɛ-dA in vascular endothelial cells. (D) Patient no. 35: many ɛ-dA-positive cells (D2) were present in hepatosarcoma and adjacent non-tumor liver tissue (D1), and mutant p53 had a positive expression in hepatosarcoma (D3), but a negative expression in adjacent non-tumor liver tissue (D3). T, HCC tissue; NT, non-tumor liver tissue. Original magnification ×200 and ×400.

The prevalence of nuclei positive for ɛ-dA in control livers was (7.9±7.5%), which was significantly increased to (29.4±15.8%) in NT and (47.6±20.6%) in T (P<0.001 and P<0.0001, respectively) (Figs. 2A and 3A2, B2 and C2). Also, the percentage of positively stained nuclei was significantly higher in T than in NT (P<0.001) (Figs. 2A and 3B2 and C2).

### Relationship between positive rate of ɛ-dA and clinicopathological features of HCC

**Figure 4:**
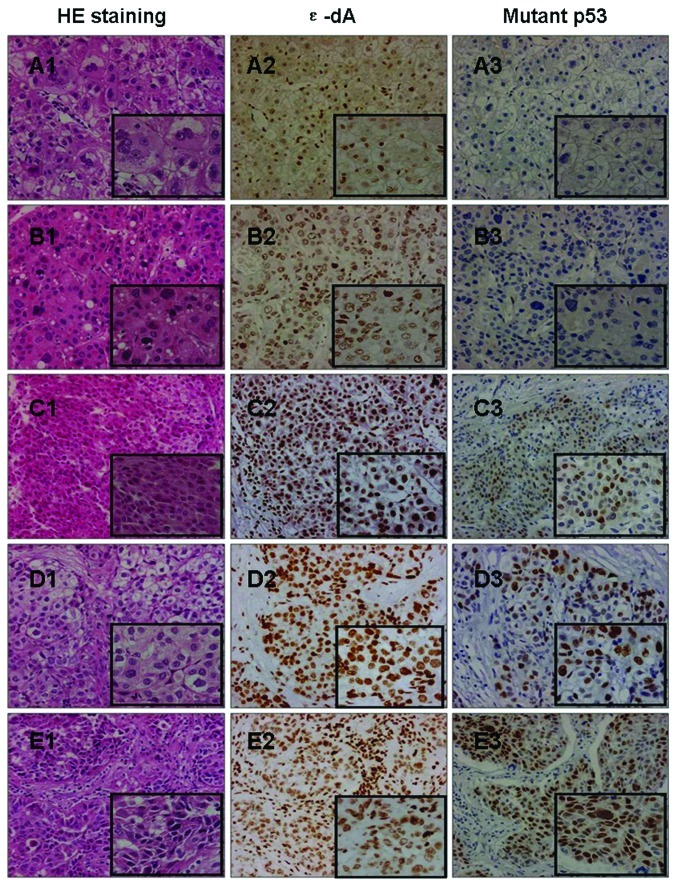
ɛ-dA and mutant p53 protein expression in different HCC tissues. (A) Patient no. 13: ɛ-dA had a slightly positive expression (A2) in well differentiated HCC (A1), but mutant p53 had a negative expression (A3). (B) Patient no. 40: ɛ-dA had little expression (B2) in well-moderately differentiated HCC (B1), but mutant p53 also had a negative expression (B3). (C) Patient no. 32: ɛ-dA had a positive expression (C2) in moderately differentiated HCC (C1), and mutant p53 also had a positive expression (C3). (D) Patient no. 38: ɛ-dA (D2) and mutant p53 (D3) had significantly higher expressions in moderately-poorly differentiated HCC (D1). (E) Patient no. 16: ɛ-dA (E2) and mutant p53 (E3) had significantly positive expressions in poorly differentiated HCC (E1). Original magnification ×200 and ×400.

We analyzed the relationship between positive rate of ɛ-dA and clinical features of HCC patients. There was no significant association between ɛ-dA positive expression and age, etiology, tumor number, metastasis, TNM stage (data not shown). On the other hand, the prevalence of ɛ-dA was positively correlated with differentiation. Group II showed a higher percentage of ɛ-dA positivity than group I (58.8±22.3 vs. 42.2±17.9%; P<0.05) (Figs. 2B and 4A2, B2, C2, D2 and E2).

### Relationship between positive rate of ɛ-dA and necroinflammatory activity and fibrosis extent of non-tumor liver tissues

**Figure 5:**
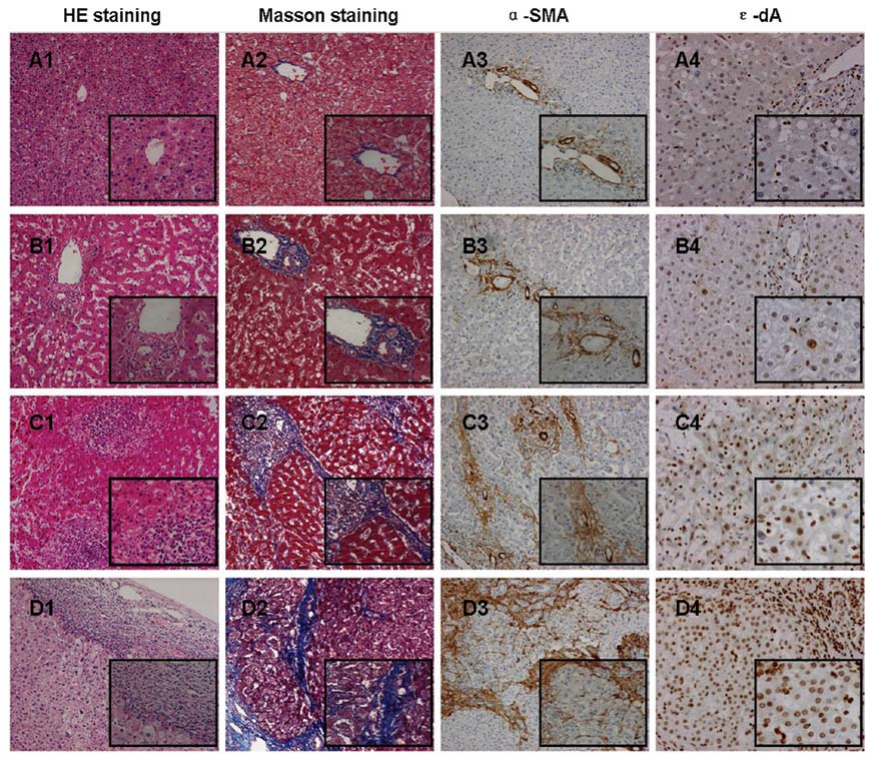
Comparison of necroinflammatory activity, the stage of fibrosis and the presence of ɛ-dA in different non-tumor liver tissues. (A) Patient no. 22: ɛ-dA had a low expression in non-tumor liver tissue which was scored as A0F1. (B) Patient no. 23: ɛ-dA also had a low expression in non-tumor liver tissue which was scored as A1F1. (C) Patient no. 26: ɛ-dA had a high expression in non-tumor liver tissue which was scored as A2F2. (D) Patient no. 20: ɛ-dA had a significantly high expression in non-tumor liver tissue which was scored as A2F3. HE, Masson and α-SMA staining: original magnification ×100 and ×200; ɛ-dA staining: original magnification ×200 and ×400.

Based on the grade of necroinflammatory activity (A), NT were subdivided into group I (A0 + A1) and group II (A2 + A3). We found the positive percentage of ɛ-dA in group I was significantly lower than in group II (21.9±11.9 vs. 39.1±15.2%; P<0.01) (Figs. 2C and 5A4, B4, C4, and D4). Based on the stage of fibrosis (F), NT were subdivided into group I (F0 + F1 + F2) and group II (F3 + F4). The prevalence of nuclei positive for ɛ-dA in group I was 21.7±12.0%, which was significantly increased to 37.1±15.6% in group II (P<0.01) (Figs. 2D and 5A4, B4, C4 and D4).

### Nuclear expression of mutant p53 protein in liver tissues

**Figure 6:**
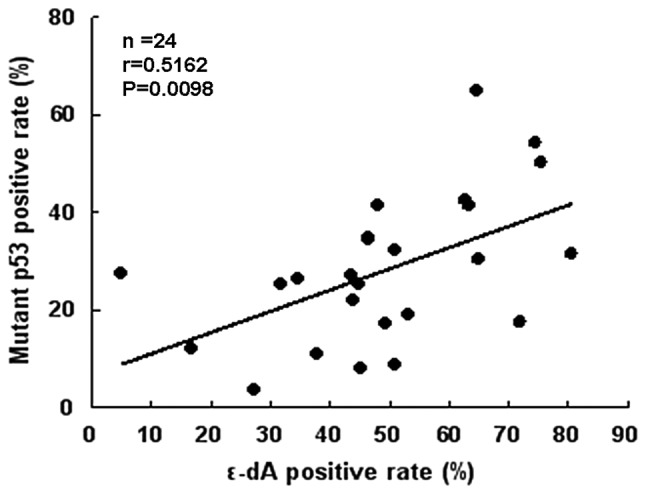
Correlation between positive rate of ɛ-dA and the mutant p53 protein in HCC tissues.

The nuclear expression of mutant p53 protein was detectable in 26 of 30 T (86.7%) whereas this was not found in any control (Fig. 3A3) or NT (Fig. 3C3). Only well (n=1) (Fig. 4A3), well-moderately (n=1) (Fig. 4B3), and moderately (n=2) differentiated HCC showed negative expression of the mutant p53 protein. The expression levels of the mutant p53 protein tended to be higher in group II compared to group I tumors (34.4±15.1 vs. 21.8±17.5%; P=0.062) (Fig. 4A3, B3, C3, D3 and E3). No significant differences in mutant p53 expression in association with age, etiology, tumor number, metastasis, TNM stage were observed (data not shown). However, the positive rate of ɛ-dA showed a significant correlation with the expression rate of the mutant p53 protein in T (n=24, r=0.5162, P<0.01) (Figs. 4 and 6).

### Positive expression of PCNA in liver tissues

**Figure 7:**
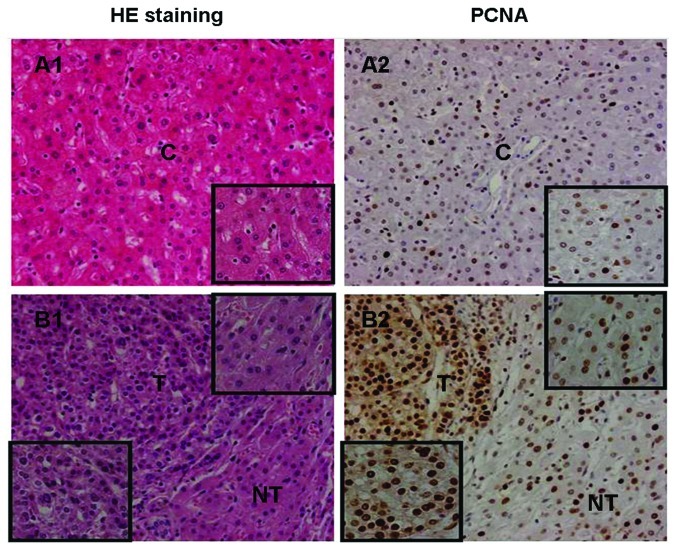
PCNA expression in different liver tissues. (A1 and A2) Patient no. 45: many PCNA-positive hepatocytes (A2) were present in the adjacent liver tissue of hemangioma (A1). (B1 and B2) Patient no. 10: many PCNA-positive cancer cells (B2) were present in T (B1), and many PCNA-positive hepatocytes (B2) were also present in adjacent NT (B1). C, control liver tissue; NT, non-tumor liver tissue; T, HCC tissue. Original magnification ×200 and ×400.

The positive rate of PCNA in T (47.6±10.9%) was significantly higher than in the adjacent NT (34.1±8.2%, P<0.0001) (Fig. 7B2) and control livers (25.8±7.7%; P<0.0001) (Fig. 7A2). The positive rate of PCNA in the NT was higher compared to the control livers (P<0.05) (Fig. 7A2 and B2). There was no significant association between PCNA positive expression and any clinicopathological feature in T or NT (data not shown).

## Discussion

In the present study, the significant decrease in the levels of T-AOC and total SOD activity in HCC tissue samples provides evidence that the antioxidant system of HCC tissue was severely impaired, and HCC tissue underwent serious oxidative stress. The prevalence of nuclei positive for ɛ-dA in HCC tissues was significantly higher compared to control and non-tumor liver tissues; it is suggested that oxidative stress could induce the excessive formation of ɛ-dA in HCC and ɛ-dA might be implicated in hepatocarcinogenesis. The poorer differentiated HCC had the lower levels of T-AOC and total SOD activity. On the contrary, the poorer differentiated HCC had the higher prevalence of ɛ-dA. Our findings are similar to a previous report that HCC tissues showed stronger immunoreactivity of 8-hydroxy-2′-deoxyguanosine (8-OHdG) which reflects oxidative DNA damage by oxidative stress than non-tumor counterparts, and the number and positive rates of 8-OHdG-stained hepatocytes was greater in poorly differentiated HCC than in well and moderately differentiated HCC (21). These findings indicated that oxidative DNA damage may be closely associated with the development of HCC.

A previous report showed that the characteristic patterns of p53 mutations were observed in liver tumors (including HCC and liver angiosarcoma) associated with vinyl chloride exposure in rats and humans. These mutations are consistent with the pro-mutagenic properties of ɛ-DNA adducts by vinyl chloride in the liver, that may be compatible with the hypothesis indicating pro-mutagenic ɛ-DNA adducts as the initiating lesions (15). One study identified the mutations of p53 in tumors induced by urethane in A/J mice, that also suggested they could arise from ɛ-DNA adducts (16). In our study, we found the positive prevalence of ɛ-dA and mutant p53 protein in HCC and hepatosarcoma tissues (Fig. 3D1-3), and the positive rate of ɛ-dA was significantly correlated with the expression rate of the mutant p53 protein in HCC tissues. This suggests that ɛ-dA may increase the incidence of p53 gene mutation in HCC tissues. Moreover, we noted that the levels of T-AOC and total SOD activity tended to be lower, but the expression levels of ɛ-dA and mutant p53 tended to be higher in poorly differentiated tumors compared to well and moderately differentiated tumors. Collectively, these findings could be explained by the observation that ROS can cause oxidative DNA damage, gene mutation or deletion, a loss of p53 function, and a defect in DNA repair capacity. This would promote genomic instability leading to oncogene activation, aberrant metabolism, mitochondrial dysfunction and a deficit in antioxidants. All these events can further increase ROS levels, leading to more DNA damage and genomic instability. Such a vicious cycle can effectively amplify oxidative stress and promote cancer development and progression (22).

The wild-type p53 protein has a crucial role in preventing oxidative damage to DNA, genetic instability, and carcinogenesis. The p53 gene is one of the most common targets for genetic alterations in HCC, being mutated and accumulated in tumor tissues. Studies *in vitro* and *in vivo* confirm that the expression of the mutant p53 protein can have a positive effect on cell growth and drive the development of different types of tumors (23,24). In our study, we found that the mutant p53 protein was expressed in most HCCs, and the positive rate of PCNA in the HCC was significantly higher than in the adjacent non-tumor and control liver tissues, indicating that the expression of mutant p53 protein has a positive effect on cell proliferation and contributes to the development of HCC. Therefore, the elevated accumulation of ɛ-dA in liver tissues may stimulate cell proliferation and promote hepatocarcinogenesis by inducing the overexpression of mutant p53 protein.

Chronic hepatitis and fibrosis/cirrhosis are the preneoplastic liver lesions that characterize the setting in which HCC most often develops. Kitada *et al* reported that 8-OHdG was observed by immunohistochemistry in patients with various forms of chronic liver diseases, and the number of positive hepatocytes was significantly correlated with the severity of chronic hepatitis activity (25). In our study, we found that ɛ-dA levels were significantly higher in non-tumor liver tissues with moderate and severe inflammation compared to those with absent and mild inflammation, and many ɛ-dA-positive hepatocytes were localized in contact with infiltrating lymphocytes in non-tumor liver tissues (Fig. 5D4). These findings can be explained by the observation that activated inflammatory cells represent a significant endogenous source of ROS that is capable of inducing DNA damage and genomic instability, and inflammatory cells may use cytokines as TNF-α to stimulate ROS accumulation in neighboring cells (26). We also found that more severe fibrosis development tended to have higher ɛ-dA levels in non-tumor liver tissues. This finding is consistent with the fact that ROS and other oxidative stress-related intermediates, released by damaged hepatocytes or activated inflammatory cells, can activate hepatic stellate cells (HSCs), which are collagen-producing cells in the liver (27). Moreover, it has been reported that activated HSCs are also an important source of endogenous ROS in liver fibrogenesis, and this is mainly associated with NADPH oxidase which is expressed in activated HSCs (28). Taken together, these findings provide evidence of a possible link between ROS-generated ɛ-dA and persistent inflammation and fibrosis in chronic liver diseases. It is possible to hypothesize that chronic inflammation and fibrosis could facilitate continuous accumulation of oxidative ɛ-DNA damage, which may partly drive chronic inflammatory and fibrotic/cirrhotic liver tissues to HCC. Therefore, measurement of ɛ-dA in liver tissue could be explored as a potential biomarker for disease progression and cancer risk assessment in patients with cancer-prone liver diseases.

Excess levels of ROS-derived ɛ-DNA adducts have been measured in the patients with inflammatory cancer-prone liver diseases. Using a sensitive immunoaffinity/^32^P-postlabeling method, elevated levels of ɛ-DNA adducts were detected in the liver of patients with primary hemochromatosis and Wilson’s disease (17). Excess ɛ-dA levels were detected in liver biopsy specimens obtained from European alcoholic liver disease (ALD) patients diagnosed with alcohol-related hepatitis, fibrosis and cirrhosis by immunohistochemistry (18). Furthermore, using an ultrasensitive and specific immunoprecipitation/high performance liquid chromatography (HPLC)-fluorescence detection method for quantifying ɛ-dA excreted in urine, significantly higher urinary ɛ-dA levels were also found in HBV and HCV-related liver disease and ALD patients compared with controls (19). These findings show that ɛ-dA may play a driving role towards hepatocarcinogenesis in cancer-prone liver diseases.

The promutagenic properties of ROS-derived ɛ-DNA adducts were established by studies in *E. coli* and in mammalian cells (29). ɛ-DNA adducts produced mainly base pair substitution mutations. ɛ-dA can lead to AT→GC transitions, to AT→TA and AT→CG transversions (30,31). ɛ-dC can cause CG→TA transitions and CG→AT transversions (32,33). *N^2^*, 3-ethenodeoxyguanosine, that is also formed *in vivo* from LPO products, can lead to GC→AT transitions (33). These miscoding properties of ɛ-bases strongly implicate them in the initiation of carcinogenesis by vinyl chloride (34), urethane (35,36) and other ɛ-adduct forming chemicals. Incorporation of a single ɛ-dA in either DNA strand of HeLa cells showed a similar miscoding frequency and was more mutagenic than 8-oxo-deoxyguanosine (37).

In conclusion, we have demonstrated that ROS-induced ɛ-dA lesions may be relevant to the severity of inflammation and fibrosis in chronic liver diseases. The elevated accumulation of ɛ-dA partly contributes to the mutant p53 protein overexpression and excessive cell proliferation, eventually leading to hepatocarcinogenesis.

## Figures and Tables

**Table I:** Clinicopathological characteristics of the 32 HCC patients.

Characteristic	Results
Gender	
Male	29
Female	3
Age (year)	
≤45	18
>45	14
Etiology	
HBV	19
HBV + alcohol	11
Unknown	2
Aflatoxin B1	
Normal	3
High	29
Tumor diameter (cm)	
≤5	5
>5	27
No. of tumors	
Single	26
Multiple	6
Pathological grade	
Well differentiated	1
Well-moderately differentiated	3
Moderately differentiated	16
Moderately-poorly differentiated	5
Poorly differentiated	7
Metastasis	
Yes	9
No	23
TNM	
I	18
II + II	14

HBV, hepatitis B virus; TNM, Tumor-Node-Metastasis.

**Table II:** Relationship between T-AOC and total SOD activity in HCC tissues with clinicopathological features.^a^

Factor	No.	T-AOC (mmol/g protein)	P-value	Total SOD activity (U/mg protein)	P-value
Age (years)			0.093		0.355
≤45	16	0.226±0.116		57.968±15.069	
>45	14	0.337±0.177		63.222±15.460	
Etiology			0.981		0.366
HBV	18	0.280±0.161		57.840±14.336	
HBV + alcohol	10	0.301±0.156		63.523±17.861	
Tumor no.			0.604		0.500
Single	24	0.290±0.172		59.843±16.676	
Multiple	6	0.225±0.035		62.728±7.526	
Differentiation^b^			0.007		0.013
Group I	19	0.324±0.134		65.531±13.298	
Group II	11	0.197±0.163		51.592±14.773	
Metastasis			0.018		0.909
Yes	8	0.388±0.176		59.898±6.588	
No	22	0.237±0.130		60.609±17.474	
TNM stage			0.098		0.786
I	17	0.241±0.147		60.342±19.438	
II + II	13	0.324±0.160		60.522±7.491	

a Data are expressed as the means ± SD.

b Based on histological grade, tumors were subdivided into group I (well, well-moderately and moderately differentiated) and group II (moderately-poorly and poorly differentiated).

T-AOC, total antioxidant capacity; SOD, superoxide dismutase; HBV, hepatitis B virus; TNM, Tumor-Node-Metastasis.

## References

[1] El-Serag HB, Rudolph KL (2007). Hepatocellular carcinoma: epidemiology and molecular carcinogenesis. Gastroenterology.

[2] Parkin DM, Bray F, Ferlay J, Pisani P (2005). Global cancer statistics, 2002. CA Cancer J Clin.

[3] Donato F, Boffetta P, Puoti M (1998). A meta-analysis of epidemiological studies on the combined effect of hepatitis B and C virus infections in causing hepatocellular carcinoma. Int J Cancer.

[4] Seitz HK, Stickel F (2006). Risk factors and mechanisms of hepatocarcinogenesis with special emphasis on alcohol and oxidative stress. Biol Chem.

[5] Severi T, Ying C, Vermeesch JR, Cassiman D, Cnops L, Verslype C, Fevery J, Arckens L, Neyts J, van Pelt JF (2006). Hepatitis B virus replication causes oxidative stress in HepAD38 liver cells. Mol Cell Biochem.

[6] Hsieh YH, Su IJ, Wang HC, Chang WW, Lei HY, Lai MD, Chang WT, Huang W (2004). Pre-S mutant surface antigens in chronic hepatitis B virus infection induce oxidative stress and DNA damage. Carcinogenesis.

[7] Qadri I, Iwahashi M, Capasso JM, Hopken MW, Flores S, Schaack J, Simon FR (2004). Induced oxidative stress and activated expression of manganese superoxide dismutase during hepatitis C virus replication: role of JNK, p38 MAPK and AP-1. Biochem J.

[8] Moriya K, Nakagawa K, Santa T, Shintani Y, Fujie H, Miyoshi H, Tsutsumi T, Miyazawa T, Ishibashi K, Horie T, Imai K, Todoroki T, Kimura S, Koike K (2001). Oxidative stress in the absence of inflammation in a mouse model for hepatitis C virus-associated hepatocarcinogenesis. Cancer Res.

[9] Wang Y, Millonig G, Nair J, Patsenker E, Stickel F, Mueller S, Bartsch H, Seitz HK (2009). Ethanol-induced cytochrome P4502E1 causes carcinogenic etheno-DNA lesions in alcoholic liver disease. Hepatology.

[10] Wu HC, Wang Q, Wang LW, Yang HI, Ahsan H, Tsai WY, Wang LY, Chen SY, Chen CJ, Santella RM (2007). Urinary 8-oxodeoxyguanosine, aflatoxin B1 exposure and hepatitis B virus infection and hepatocellular carcinoma in Taiwan. Carcinogenesis.

[11] Starley BQ, Calcagno CJ, Harrison SA (2010). Nonalcoholic fatty liver disease and hepatocellular carcinoma: a weighty connection. Hepatology.

[12] Toyokuni S, Okamoto K, Yodoi J, Hiai H (1995). Persistent oxidative stress in cancer. FEBS Lett.

[13] Bartsch H, Nair J (2006). Chronic inflammation and oxidative stress in the genesis and perpetuation of cancer: role of lipid peroxidation, DNA damage, and repair. Langenbecks Arch Surg.

[14] el Ghissassi F, Barbin A, Nair J, Bartsch H (1995). Formation of 1, *N**^6^*-ethenoadenine and 3, *N**^4^*-ethenocytosine by lipid peroxidation products and nucleic acid bases. Chem Res Toxicol.

[15] Barbin A, Froment O, Boivin S, Marion MJ, Belpoggi F, Maltoni C, Montesano R (1997). p53 gene mutation pattern in rat liver tumors induced by vinyl chloride. Cancer Res.

[16] Horio Y, Chen A, Rice P, Roth JA, Malkinson AM, Schrump DS (1996). Ki-ras and p53 mutations are early and late events, respectively, in urethane-induced pulmonary carcinogenesis in A/J mice. Mol Carcinog.

[17] Nair J, Carmichael PL, Fernando RC, Phillips DH, Strain AJ, Bartsch H (1998). Lipid peroxidation-induced etheno-DNA adducts in the liver of patients with the genetic metal storage disorders Wilson’s disease and primary hemochromatosis. Cancer Epidemiol Biomarkers Prev.

[18] Frank A, Seitz HK, Bartsch H, Frank N, Nair J (2004). Immunohistochemical detection of 1, *N**^6^*-ethenodeoxyadenosine in nuclei of human liver affected by diseases predisposing to hepatocarcinogenesis. Carcinogenesis.

[19] Nair J, Srivatanakul P, Haas C, Jedpiyawongse A, Khuhaprema T, Seitz HK, Bartsch H (2010). High urinary excretion of lipid peroxidation-derived DNA damage in patients with cancer-prone liver diseases. Mutat Res.

[20] Desmet VJ, Gerber M, Hoofnagle JH, Manns M, Scheuer PJ (1994). Classification of chronic hepatitis: diagnosis, grading and staging. Hepatology.

[21] Ichiba M, Maeta Y, Mukoyama T, Saeki T, Yasui S, Kanbe T, Okano J, Tanabe Y, Hirooka Y, Yamada S, Kurimasa A, Murawaki Y, Shiota G (2003). Expression of 8-hydroxy-2′-deoxyguanosine in chronic liver disease and hepatocellular carcinoma. Liver Int.

[22] Trachootham D, Alexandre J, Huang P (2009). Targeting cancer cells by ROS-mediated mechanisms: a radical therapeutic approach?. Nat Rev Drug Discov.

[23] Bossi G, Lapi E, Strano S, Rinaldo C, Blandino G, Sacchi A (2006). Mutant p53 gain of function: reduction of tumor malignancy of human cancer cell lines through abrogation of mutant p53 expression. Oncogene.

[24] Shaulsky G, Goldfinger N, Rotter V (1991). Alterations in tumor development in vivo mediated by expression of wild type or mutant p53 proteins. Cancer Res.

[25] Kitada T, Seki S, Iwai S, Yamada T, Sakaguchi H, Wakasa K (2001). In situ detection of oxidative DNA damage, 8-hydroxydeoxyguanosine, in chronic human liver disease. J Hepatol.

[26] Grivennikov SI, Greten FR, Karin M (2010). Immunity, inflammation, and cancer. Cell.

[27] Novo E, Parola M (2008). Redox mechanisms in hepatic chronic wound healing and fibrogenesis. Fibrogenesis Tissue Repair.

[28] Bataller R, Schwabe RF, Choi YH, Yang L, Paik YH, Lindquist J, Qian T, Schoonhoven R, Hagedorn CH, Lemasters JJ, Brenner DA (2003). NADPH oxidase signal transduces angiotensin II in hepatic stellate cells and is critical in hepatic fibrosis. J Clin Invest.

[29] Bartsch H, Barbin A, Marion MJ, Nair J, Guichard Y (1994). Formation, detection, and role in carcinogenesis of ethenobases in DNA. Drug Metab Rev.

[30] Basu AK, Wood ML, Niedernhofer LJ, Ramos LA, Essigmann JM (1993). Mutagenic and genotoxic effects of three vinyl chloride-induced DNA lesions: 1, *N**^6^*-ethenoadenine, 3, *N**^4^*-ethenocytosine, and 4-amino-5-(imidazol-2-yl)imidazole. Biochemistry.

[31] Pandya GA, Moriya M (1996). 1, *N**^6^*-ethenodeoxyadenosine, a DNA adduct highly mutagenic in mammalian cells. Biochemistry.

[32] Palejwala VA, Rzepka RW, Simha D, Humayun MZ (1993). Quantitative multiplex sequence analysis of mutational hot spots. Frequency and specificity of mutations induced by a site-specific ethenocytosine in M13 viral DNA. Biochemistry.

[33] Moriya M, Zhang W, Johnson F, Grollman AP (1994). Mutagenic potency of exocyclic DNA adducts: marked differences between *Escherichia coli* and simian kidney cells. Proc Natl Acad Sci USA.

[34] Cheng KC, Preston BD, Cahill DS, Dosanjh MK, Singer B, Loeb LA (1991). The vinyl chloride DNA derivative *N**^2^*, 3-ethenoguanine produces G→A transitions in *Escherichia coli*. Proc Natl Acad Sci USA.

[35] Barbin A, Bartsch H, Leconte P, Radman M (1981). Studies on the miscoding properties 1, *N**^6^*-ethenoadenine and 3, *N**^4^*-ethenocytosine, DNA reaction products of vinyl chloride metabolites, during in vitro DNA synthesis. Nucleic Acids Res.

[36] Miller JA, Miller EC (1983). The metabolic activation and nucleic acid adducts of naturally-occurring carcinogens: recent results with ethyl carbamate and the spice flavors safrole and estragole. Br J Cancer.

[37] Levine RL, Yang IY, Hossain M, Pandya GA, Grollman AP, Moriya M (2000). Muta-genesis induced by a single 1, *N**^6^*-ethenodeoxyadenosine adduct in human cells. Cancer Res.

